# Environmentally prevalent polycyclic aromatic hydrocarbons can elicit co-carcinogenic properties in an in vitro murine lung epithelial cell model

**DOI:** 10.1007/s00204-017-2124-5

**Published:** 2017-11-23

**Authors:** Alison K. Bauer, Kalpana Velmurugan, Sabine Plöttner, Katelyn J. Siegrist, Deedee Romo, Peter Welge, Thomas Brüning, Ka-Na Xiong, Heiko U. Käfferlein

**Affiliations:** 1 Department of Environmental and Occupational Health, Colorado School of Public Health, University of Colorado Anschutz Medical Campus, Mailstop V-20, Rm 3125, 12850 E. Montview Blvd, Aurora, CO 80045 USA; 2 Institute for Prevention and Occupational Medicine of the German Social Accident Insurance, Institute of the Ruhr-University Bochum (IPA), 44789 Bochum, Germany

**Keywords:** Polycyclic aromatic hydrocarbons, DNA adducts, Gap junctions, Benzo[a]pyrene, Fluoranthene, 1-Methylanthracene

## Abstract

Low molecular weight (LMW) polycyclic aromatic hydrocarbons (PAH) are the most abundant PAHs environmentally, occupationally, and are in cigarette smoke; however, little is known about their carcinogenic potential. We hypothesized that LMW PAHs act as co-carcinogens in the presence of a known carcinogen (benzo[*a*]pyrene (B[*a*]P)) in a mouse non-tumorigenic type II cell line (C10 cells). Gap junctions are commonly suppressed and inflammation induced during tumor promotion, while DNA-adduct formation is observed during the initiation stage of cancer. We used these endpoints together as markers of carcinogenicity in these lung adenocarcinoma progenitor cells. LMW PAHs (1-methylanthracene and fluoranthene, 1–10 µM total in a 1:1 ratio) were used based on previous studies as well as B[*a*]P (0–3 µM) as the classic carcinogen; non-cytotoxic doses were used. B[*a*]P-induced inhibition of gap junctional intercellular communication (GJIC) was observed at low doses and further reduced in the presence of the LMW PAH mixture (*P* < 0.05), supporting a role for GJIC suppression in cancer development. Benzo[*a*]pyrene diol-epoxide (BPDE)-DNA adduct levels were significantly induced in B[*a*]P-treated C10 cells and additionally increased with the LMW PAH mixture (*P* < 0.05). Significant increases in cyclooxygenase (*Cox-2*) were observed in response to the B[*a*]P/LMW PAH mixture combinations. DNA adduct formation coincided with the inhibition of GJIC and increase in *Cox-2* mRNA expression. Significant cytochrome p4501b1 increases and connexin 43 decreases in gene expression were also observed. These studies suggest that LMW PAHs in combination with B[*a*]P can elicit increased carcinogenic potential. Future studies will further address the mechanisms of co-carcinogenesis driving these responses.

**Electronic supplementary material:**

The online version of this article (10.1007/s00204-017-2124-5) contains supplementary material, which is available to authorized users.

## Introduction

Polycyclic aromatic hydrocarbons (PAHs) are a class of environmentally ubiquitous toxicants in water, air, and soil as well as in occupational settings (ATSDR 2005). Epidemiological studies demonstrated increased lung cancer and other pulmonary disease risks are associated with PAHs from environmental and occupational exposures (IARC 2012a, b). PAHs are formed from the incomplete combustion of organic material containing two or more fused benzene rings (ATSDR 2005) and are found in high amounts in firsthand, secondhand, and thirdhand smoke from cigarettes, at hazardous waste sites, oil production sites, and are components of diesel exhaust (ATSDR 2005; Lee et al. 2010). PAHs also attach to particulate matter (PM), both PM_2.5_ and PM_10_, and elevated PAH concentrations have been observed in mega-cities compared to rural and undeveloped natural environments (Hong et al. 2016; Zhang et al. 2016). Thus, based on the numerous potential exposures, PAHs are an international public health concern.

The classic reference PAH used to evaluate the toxicity of all PAHs is benzo[*a*]pyrene (B[*a*]P), one of the class of PAHs called high molecular weight (HMW) PAHs due to a ring structure ≥ 5 and molecular weight > 207 g/mol. B[*a*]P is a known carcinogen classified by the International Agency for Research on Cancer (IARC) as a group 1 carcinogen (IARC 2010), while the majority of other HMW PAHs are largely classified in the group 2A (probably carcinogenic) or 2B (possibly carcinogenic) categories (IARC 2010). However, of the 16 U.S.E.P.A. priority PAHs, 8 are low molecular weight (< 206 g/mol) and considered non-genotoxic, with the exception of naphthalene (IARC 2010; U.S.E.P.A. 2002). These low molecular weight (LMW) PAHs are also classified under IARC group 3 and are therefore “not classifiable as to their carcinogenicity to humans” (IARC 2010).

Several studies demonstrated that LMW PAHs are more prevalent in secondhand versus firsthand cigarette smoke. Thirdhand smoke, components of secondhand smoke that persist on walls, and other areas in indoor environments, contain PAHs, among other components (Schick et al. 2013). Fluoranthene (Flthn), a LMW PAH, is present in relatively high concentrations in thirdhand smoke (Schick et al. 2013). In addition, fluoranthene and other LMW PAHs are also prevalent in diesel exhaust (DE), and were far more abundant than HMW species at oil production sites (ATSDR 2005). There is also international concern for exposure to LMW PAHs and their impacts on human health based on few environmental regulations (Obiri et al. 2011; Oliveira et al. 2011). For example, fluoroanthene was identified as one of the higher concentration PAHs in the air in Beijing, China (Wu et al. 2014). Additionally, LMW PAHs are found in food (Guillen et al. 2007), sediment (particularly methylanthracenes) (Vondracek et al. 2007), and occupational exposures above background levels occur worldwide in industries involving coal tar (i.e., roofers, chemical oil), among others (ATSDR 2005; IARC 2010). Occupational studies on asphalt workers, for example, demonstrated that dermal 2–4 ring PAH levels averaged > 60 µg/day (Fustinoni et al. 2010) and LMW PAH are also taken up in various other occupational settings such as in the coal, coke and steel industry (Marczynski et al. 2009; Pesch et al. 2007; Talaska et al. 2014). Thus, the many sources of these PAHs suggest that humans are exposed through multiple routes; however respiratory and specifically co-carcinogenic effects of these specific LMW PAHs have not been established.

Evaluating multiple endpoints to identify appropriate biomarkers of disease, specifically cancer, are important to improve future risk assessments and policy changes in how LMW PAHs are regulated. Thus, in these studies we investigated a pulmonary cell line to determine if lung specific benzo[*a*]pyrene diol-epoxide (BPDE)-DNA adducts were formed following B[*a*]P exposure and if BPDE adduct formation is influenced by LMW PAHs. We also evaluated gap junctional intercellular communication (GJIC), another well-established endpoint inhibited during early stages of tumor development (e.g., tumor promotion)(Trosko and Upham 2010). In several recent studies, we showed LMW PAHs inhibited gap junctional activity and the primary pulmonary connexin protein that forms the gap junction channel (connexin 43) (Osgood et al. 2013, 2017). Lastly, we assessed mRNA expression of a *Cyp1a1* and *Cyp1b1*, two important enzymes involved in the activation of B[*a*]P, and of a previously identified inflammatory mediator called cyclooxygenase 1 (*Cox-2*) that is involved in prostaglandin production and known downstream inflammatory and proliferative effects (Bazzani et al. 2017). *Cox-2* gene expression was also significantly induced following acute LMW binary PAH exposure (Osgood et al. 2013, 2017). Collectively we hypothesized that LMW PAHs act as co-carcinogens in the presence of a known carcinogen (B[a]P) in a mouse alveolar type II cell line (C10 cells). Because these type II cells are a progenitor cell for lung adenocarcinoma (ADC), the most prevalent type of lung cancer, we used this model as an organ-specific surrogate to demonstrate the effects of these PAH combinations in lung.

## Materials and methods

### Chemicals

**Fig. 1:**
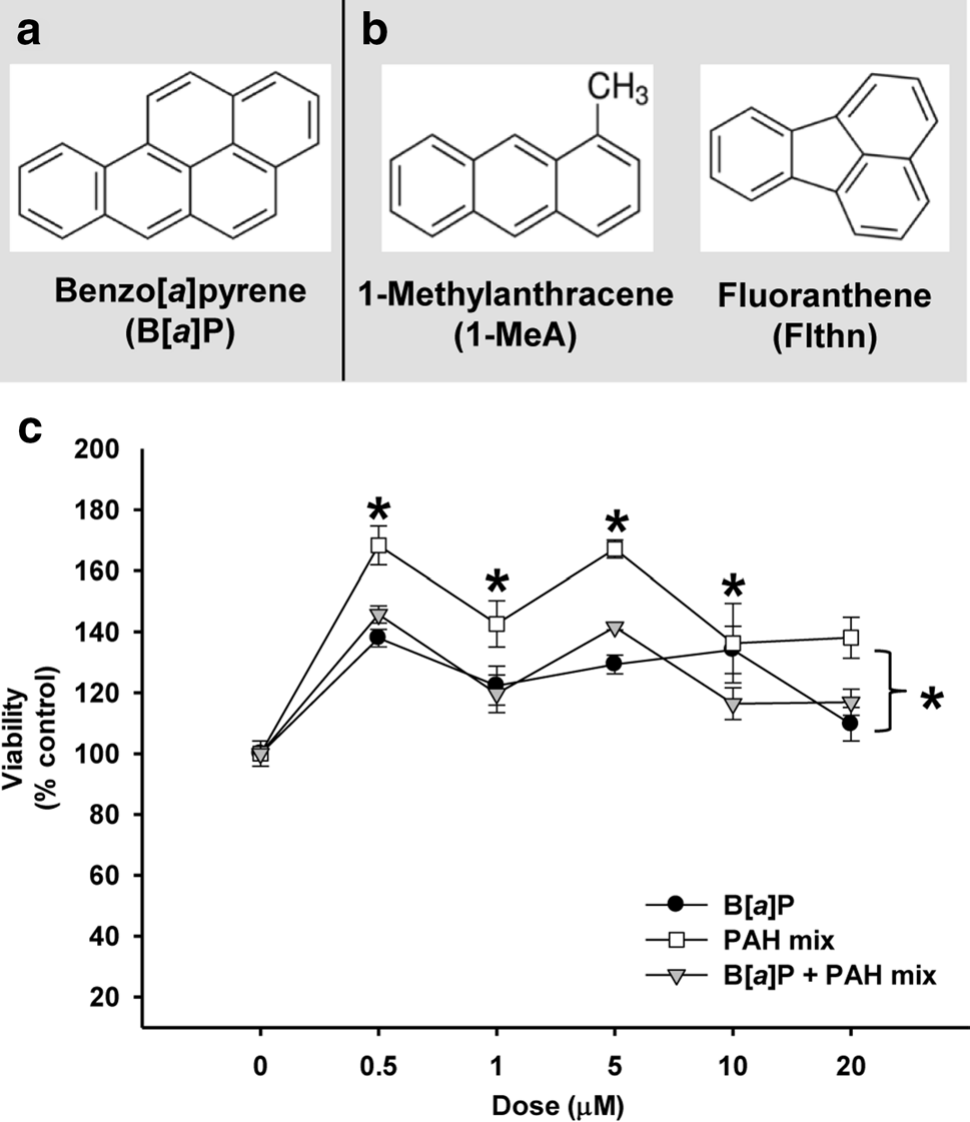
Benzo[*a*]pyrene (B[*a*]P), a LMW binary PAH mixture [1-methylanthracene (1-MeA) and fluoranthene (Flthn)], and combinations of the two are not cytotoxic to C10 cells. **a** Chemical structure of HMW PAH B[*a*]P and **b** LMW PAHs 1-MeA and Flthn studied. **c** Cytotoxicity for PAH exposure in C10 cells following 24 h exposure using the MTS assay. Mean ± SEM presented; *n* = 3 per treatment; repeated twice. *P < 0.05 treatments are significantly different than control (0; DMSO)

Fluoranthene (Flthn; purity 97.2%) was purchased from AccuStandard (New Haven, CT), benzo[*a*]pyrene (B[*a*]P; purity ≥ 96%) from Sigma-Aldrich (St. Louis, MO, USA) and 1-methylanthracene (1-MeA; purity 99.5%) from Crescent Chemical (Islandia, NY, USA). Chemical structures for these PAHs are depicted in Fig. 1a, b. Dimethyl sulfoxide (DMSO) and Lucifer Yellow were purchased from Sigma-Aldrich. All PAH stock solutions for treatment were prepared in DMSO. B[*a*]P-tetrol I-1 was obtained from the Biochemical Institute for Environmental Carcinogens, Großhansdorf, Germany.

Methanol ROTISOL^®^ (HPLC gradient grade) was purchased from Carl Roth GmbH (Karlsruhe, Germany).

### Cell culture

The C10 cell line was obtained from Dr. Lori Nield (University of Colorado) and is an immortalized, non-transformed alveolar type II cell line originally derived from a BALB mouse (Malkinson et al. 1997). These cells are one of the best models for type II cells which are a progenitor/stem cell type for lung adenocarcinoma and have been extensively reviewed (Malkinson et al. 1997). C10 cells exhibit normal gap junctional communication (Malkinson et al. 1997) and were characterized for the acute (0–24 h) LMW PAH effects in several previous publications (Osgood et al. 2017, 2013). Cells (passage < 20) were maintained in CMRL 1066 media (Gibco, Thermo Fisher Scientific) containing 10% FBS and 1% glutamate in a humidified atmosphere at 37 °C, 5% CO_2_, and 95% air (Osgood et al. 2013). Cells were grown to confluence (2–3 days) in 35 mm diameter (scaple-loaded/dye-transfer assay (SL/DT) for GJIC, RNA extraction) or 60 mm diameter (protein extraction) tissue culture dishes, or in 175 cm^2^ flasks (DNA isolation, anti-BPDE adduct analysis) (Greiner, Cell Star, USA Scientific, Ocala, FL, USA). At confluence, cells were serum-deprived for 24 h prior to treatment with the single B[*a*]P, the binary PAH mixture of 1-MeA and Flthn, or the combination of B[*a*]P + binary PAH mixture for all endpoints measured except DNA adducts, based on previous studies (Osgood et al. 2013, 2017; Plöttner et al. 2016). The binary PAH mixture was previously used and represents two PAHs common in secondhand smoke, PM exposures, and in sediments (Lee et al. 2010; Osgood et al. 2017; Vondracek et al. 2007). DMSO concentrations (< 0.01%) did not elicit cytotoxicity to the cells; no differences between the DMSO and media control were observed.

### Cytotoxicity

Cytotoxicity was evaluated using the CellTiter 96 AQueous One Solution Cell Viability assay (MTS assay, Promega, Madison, WI) following manufacturer’s instructions. Cells were grown to confluence in 96 well tissue culture plates (Greiner) and serum-deprived as described above.

### DNA isolation

C10 cells were exposed to B[*a*]P (0.3, 1 and 3 µM), 10 µM of the binary PAH mix, or the combination of 1 µM B[a]P + PAH mix (0.01, 0.1, 1, 5 or 10 µM, depending on the experiments) for 24 h in three separate experiments. Untreated and DMSO-treated cells were included as negative controls. Cells were harvested and their pellets stored at − 80 °C. DNA was isolated from individual samples (5-mL aliquots, each containing ~ 9–16 × 10^6^ cells) using the QIAamp® DNA Blood Maxi Kit (Qiagen, Hilden, Germany) according to the manufacturer’s instructions. The first two eluates with nucleic acids were pooled and treated at 37 °C with DNase-free RNase A (Qiagen, Hilden, Germany; final concentration 20 µg/mL) and RNase T1 (Sigma-Aldrich, Taufkirchen, Germany; final concentration 10 µg/mL) in TE buffer (10 mM Tris–HCl, 1 mM Na2-EDTA; pH 8.0). After 30-min incubation, NaCl (final concentration 100 mM) and two volumes of 95% ethanol were added. Samples were vigorously mixed and allowed to stand at RT for 10 min prior to centrifugation (13,000*g*, 2 min, RT). Pelleted DNA was dissolved in ultrapure water. Specific content of DNA and remains of RNA were determined using Qubit® dsDNA BR and Qubit® RNA HS assay kits with a Qubit® 3.0 fluorimeter according to the manufacturer’s instructions (Life Technologies, Darmstadt, Germany). The remaining RNA content was ≤ 5%.

### Analysis of anti-BPDE-DNA adducts

**Fig. 2:**
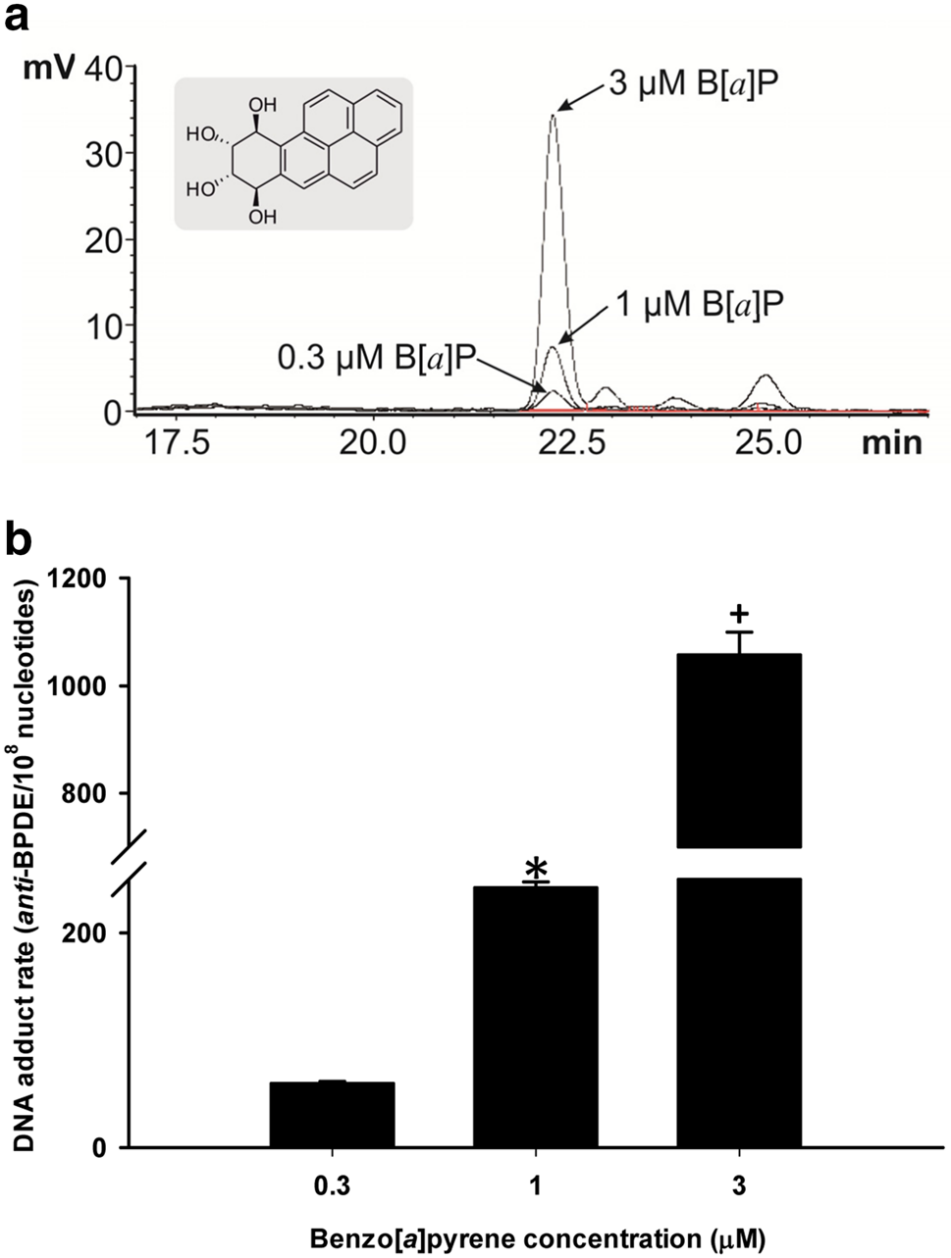
B[*a*]P dose dependent response for anti-B[*a*]P DNA adduct formation in C10 cells. **a** Representative overlay chromatograms from HPLC analysis of B[a]P-tetrol I-1 (structure, inset) in isolated DNA of B[a]P-treated C10 cells after acidic hydrolysis. Cells were incubated for 24 h with 0.3, 1 or 3 µM B[a]P and 31.7, 25.5 or 26.4 µg DNA were applied. **b** Mean ± SEM of the DNA adduct rate; *n* = 3. *P < 0.001 compared to 0.3 dose; + *P* < 0.001 compared to other doses

Analysis of *anti*-BPDE-DNA adducts was carried out in terms of the B[*a*]P-specific analyte (±)-*r*-7,*t*-8,*t*-9,*c*-10-tetrahydroxy-7,8,9,10-tetrahydro-B[*a*]P (B[*a*]P-tetrol I-1, Fig. 2) after acidic hydrolysis of DNA. *Anti*-BPDE-DNA adducts were determined as previously described by Alexandrov et al. (1992) and with minor modifications as described by Mensing et al. (2005). We used 0.1 N HCl, 90 °C, and 3 h for acidic hydrolysis followed by high-performance liquid chromatography with fluorescence detection (HPLC-FLD, Shimadzu, Duisburg, Germany). Six hundred μL of (1) samples containing purified DNA, (2) B[*a*]P-tetrol I-1 standard (0.0095-1.52 μg/L) in combination with calf-thymus DNA (Sigma, Taufkirchen, Germany) or (3) a blank [55% (v/v)] methanol in water in combination with calf-thymus DNA were injected. Separation of analytes was performed on a RP C18 column (Gromsil 120 ODS-3CP, 5 µm, 250 × 4.6 mm, Dr. Maisch, Ammerbuch, Germany); whereas fluorescence detection was carried out at 344 nm (ex.)/398 nm (em.). The data represents the mean values and standard deviations from three separate incubation experiments.

### Scalpel-loaded dye-transfer assay (SL/DT)

Cells were grown to confluence before three cuts were made with a steel scalpel blade in the presence of Lucifer Yellow (1 mg/ml in PBS), following the method described by Upham et al. (2016). Briefly, the Lucifer Yellow was allowed to transfer through gap junctions for 3 min and then cells were fixed with 4% formalin. The area of dye spread was imaged with an Eclipse Ti-S microscope at 100X. Images were captured with a DS-QiMc camera (Nikon Instruments, Melville, NY) and quantified using ImageJ software (http://imagej.nih.gov/ij/). Area of dye spread was quantified by comparing B[*a*]P, the binary PAH mixture, and B[*a*]P + the LMW binary PAH mixture treated cells to DMSO control for the final fraction of control (FOC) percentages. For the SL/DT assays, three cut lines were analyzed per dish, 3 dishes per treatment. These experiments were all repeated three times.

### Quantitative reverse transcriptase polymerase chain reaction (qRT-PCR)

One microgram of total RNA was reverse transcribed to cDNA (Bauer et al. 2011; Bauer et al. 2016; Osgood et al. 2017) and amplified with gene-specific primers labeled with SYBR Green master mix (Kappa Biosystems, Wilmington, MA) using an Eppendorf Mastercycler ep Realplex (Eppendorf, Hauppauge, NY). Samples were normalized to the expression of 18S rRNA using the comparative CT method (Bauer et al. 2017). Sequences for the primers (*Cyp1a1, Cyp1b1, Gja1, Cox-2*) can be found in Online Resource 1. These experiments were all repeated three times.

### Connexin 43 (Cx43) immunoblots

Twenty percent SDS containing protease inhibitor (Protease Inhibitor Cocktail 100×, Sigma-Aldrich, St Louis, MO, USA) and phosphatase inhibitor (Halt Phosphatase Inhibitor Cocktail 100×, Thermo Fisher Scientific, Waltham, MA, USA) was used to extract proteins from the cells, similar to Osgood et al. (2013 and 2017). Fifteen µg of protein was separated on 12.5% SDS page gels and transferred to a polyvinylidene fluoride (PVDF) membrane (Millipore). Anti-mouse Cx43 antibody (Millipore, Bilerica, MA, cat# MAB3068; 1:1000) and anti-mouse β-Actin antibody (Sigma-Aldrich, St. Louis, MO, USA, cat# A1978; 1:1000) were used following previous studies (Osgood et al. 2017, 2013). Proteins were visualized via Odyssey Imaging system (Licor, Lincoln, NE, USA) and quantified by densitometry using the BioRad Quantity One Software (Bio-Rad, Hercules, CA, USA).

### Statistics

SigmaPlot (12.3) software (SYSTAT, San Jose, CA, USA) or GraphPad Prism (La Jolla, CA) was used for all graphs and statistical analyses; *P* < 0.05 was considered statistically significant. All data are presented as the group mean ± standard error of the mean (SEM). For all studies, experiments were repeated three times. ANOVA was used for all analyses followed by Student-Newman Keuls for *a posteriori* comparison of means.

## Results

### Cytotoxicity of tested PAH in lung epithelial cells

Cytotoxicity was evaluated in the C10 cells in response to B[*a*]P at multiple concentrations (0, 0.5, 1.0, 5.0, 10.0, 20.0 µM) from 24 h (Fig. 1c) to 48 h (data not shown). B[*a*]P at these concentrations did not elicit any toxicity at either the 24 or 48 h time point. Increases in cell density were observed suggesting proliferation. Similar responses were also observed for the LMW binary PAH mixture at the same concentrations as well as the combination of B[*a*]P and the LMW binary PAH mixture at these concentrations (Fig. 1c). Therefore, these doses of PAHs are not cytotoxic to the C10 cells and potentially induce significant proliferative responses following 24 and 48 h exposures, to be evaluated in the future.

### DNA adduct formation

**Fig. 3:**
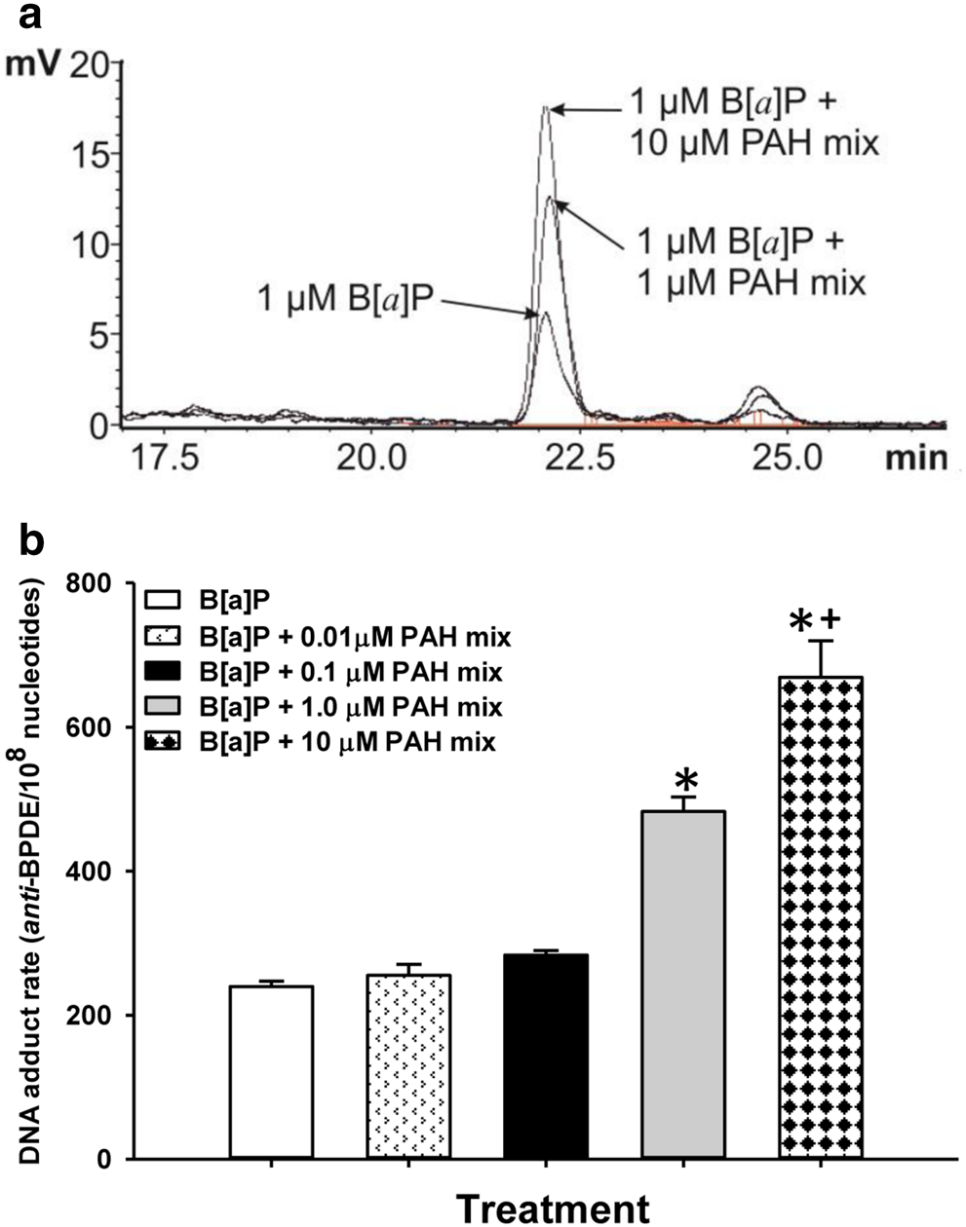
Anti-B[*a*]P DNA adduct formation is increased in the presence of a LMW binary PAH mixture in C10 cells. **a** Representative overlay chromatograms from HPLC analysis of B[a]P-tetrol I-1 in isolated DNA (24.7, 25.9 or 21.7 µg) of C10 cells treated for 24 h with 1 µM B[*a*]P or 1 µM B[*a*]P in combination with 1 or 10 µM of LMW binary PAH mixture (PAH mix) after acid hydrolysis. **b** Mean ± SEM of the DNA adduct rate of C10 cells treated with 1 µM B[*a*]P or 1 µM B[*a*]P in combination with several concentrations of LMW binary PAH mix; *n* = 3. *P < 0.001 compared to other doses; ^+^ *P* < 0.001 compared to B[*a*]P + 1 µM PAH mix

The lack of toxicity that were observed with B[*a*]P and LMW PAHs align with the enhanced numbers of anti-B[*a*]P DNA adducts that were observed in these studies. B[*a*]P elicits a significant dose dependent increase in DNA adduct formation in the C10 cells following 24 h of exposure, with a > 16-fold increase in adduct formation between the lowest (0.3 µM) and highest doses (3.0 µM) tested (Fig. 2). We then chose the 1 µM B[*a*]P dose for the combination studies to start with a dose that elicited fewer adducts alone (Fig. 3). When 1 µM B[*a*]P is compared to the combinations of B[*a*]P with increasing concentrations (0.01, 0.1, 1.0, 10.0 µM) of the LMW binary PAH mixture consisting of Flthn and 1-MeA in a 1:1 ratio, the combinations led to significant increases in adduct formation compared to B[a]P alone at 1.0 and 10.0 µM concentrations of LMW binary PAH mixture (Fig. 3). These results indicate that LMW PAHs can influence B[*a*]P responsiveness in a non-transformed mouse lung epithelial cell line.

### Dysregulation of gap junctional intercellular communication (GJIC)

**Fig. 4:**
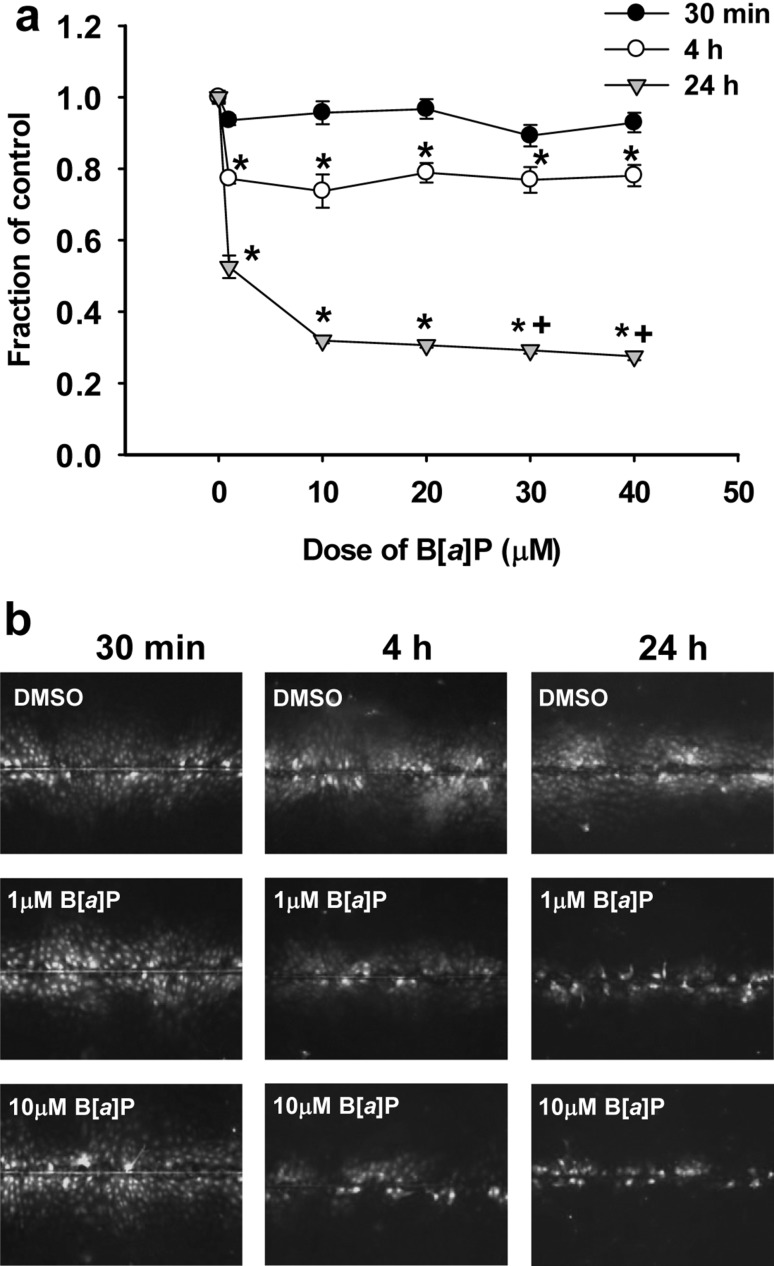
Gap junction activity is significantly reduced following B[*a*]P exposure in a dose and time dependent manner. **a** Graphical representation of GJIC activity changes determined via SL/DT assays at 30 min, 4 and 24 h of treatment with different concentrations of B[*a*]P. Mean ± SEM presented; *n* = 3, repeated twice. *P < 0.05 compared to DMSO control (0); +*P* < 0.05 compared to all other doses. **b** Representative images of GJIC activity changes at three time points in response to B[*a*]P treatment in the C10 cells using the SL/DT assay

**Fig. 5:**
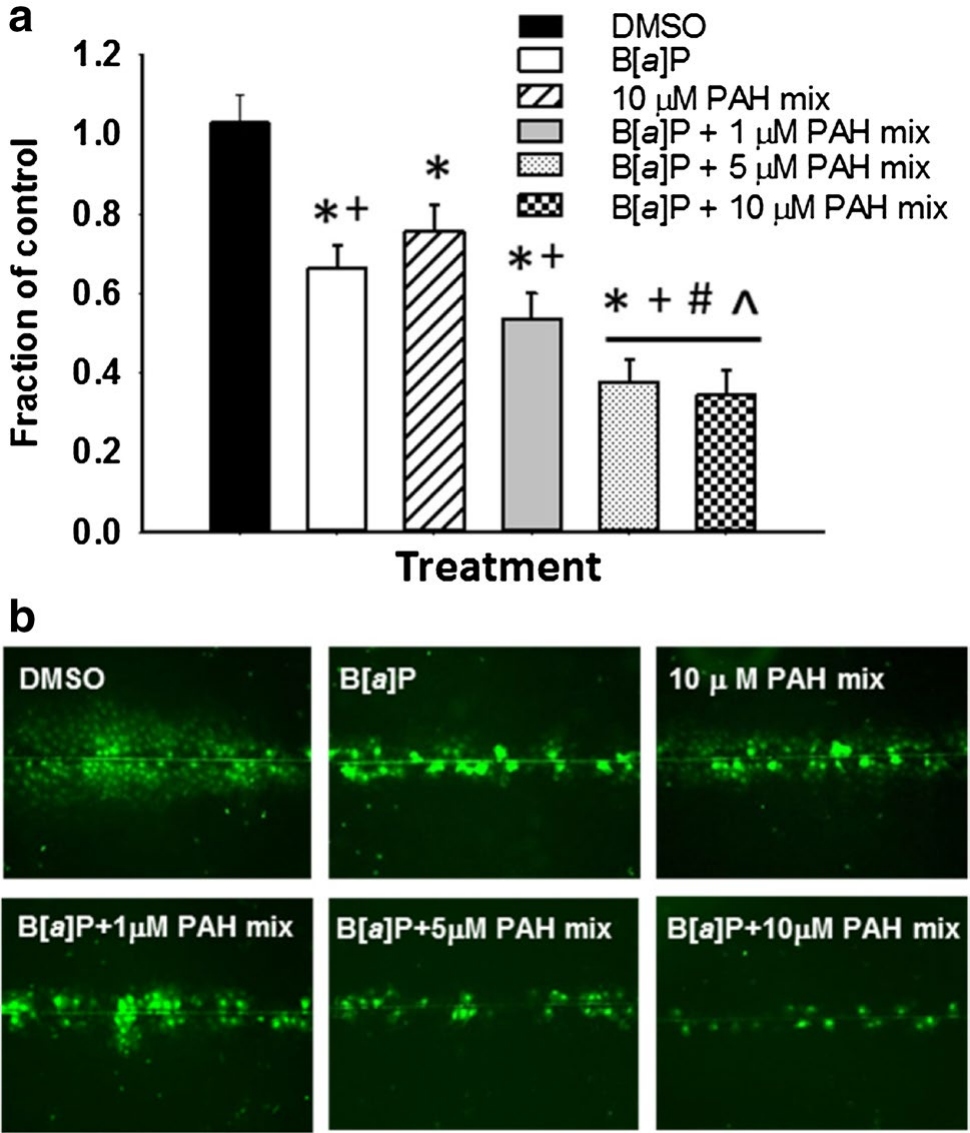
The combination of B[*a*]P and the LMW binary PAH mixture significantly reduced gap junction activity in the C10 cells. **a** Cells were treated with 1 µM B[*a*]P, 10 µM LMW binary PAH mixture, or combinations of 1µM B[*a*]P and LMW binary PAH mixture (PAH mix) at several doses (1, 5, and 10 µM) for 24 h followed by SL/DT assays for GJIC activity. Mean ± SEM presented; *n* = 3, repeated twice. *P < 0.05 compared to DMSO control; +, p < 0.05 compared to 10 µM LMW binary PAH mix; #*P* < 0.05 compared to B[*a*]P; ^*P* < 0.05 compared to B[*a*]P + 1 µM LMW binary PAH mix. **b** Depiction of GJIC activity changes in response to B[*a*]P plus combinations with the LMW binary PAH mixture in the C10 cells using the SL/DT assay

B[*a*]P alone was first evaluated to determine its effects on GJIC dysregulation at multiple concentrations (0, 1, 10, 20, 30, 40 µM) (Fig. 4) at several time points (30 min, 4 h, and 24 h). These times were chosen based on previous studies in our laboratories demonstrating reduced GJIC with the single and binary mixtures of LMW PAHs (Osgood et al. 2013, 2017). We observed significant reductions in GJIC activity following 4 and 24 h exposures; however, the extent of this dysregulation was significantly greater and more apparent at 24 h (Fig. 4). Based on these results and the DNA adduct findings at 24 h of exposure, we then investigated the influence of the LMW binary PAH mixture on B[*a*]P-induced GJIC dysregulation. We previously demonstrated that Flthn, 1-MeA and the binary PAH mixture of these two PAHs dysregulates GJIC from 15 min to 24 h (Osgood et al. 2017), thus we do not repeat those studies herein, but examine additional combinations with B[*a*]P exposure (Fig. 5). One micromolar B[*a*]P or 10 µM LMW binary PAH mixture significantly reduced GJIC by 20–40%; however, in the presence of increasing concentrations of LMW binary PAH mixture, the level GJIC inhibition exceeded 50%, and above 60% for the two highest combinations (B[a]P and both 5 and 10 µM LMW binary PAH mixture; *P* < 0.05). Thus, the combinations of a known carcinogen (B[a]P) and the addition of LMW PAHs significantly reduced GJIC activity in the pulmonary C10 cells (Fig. 5), similar to increased numbers of DNA adducts formed.

### Changes in Cx43 mRNA and protein expression

**Fig. 6:**
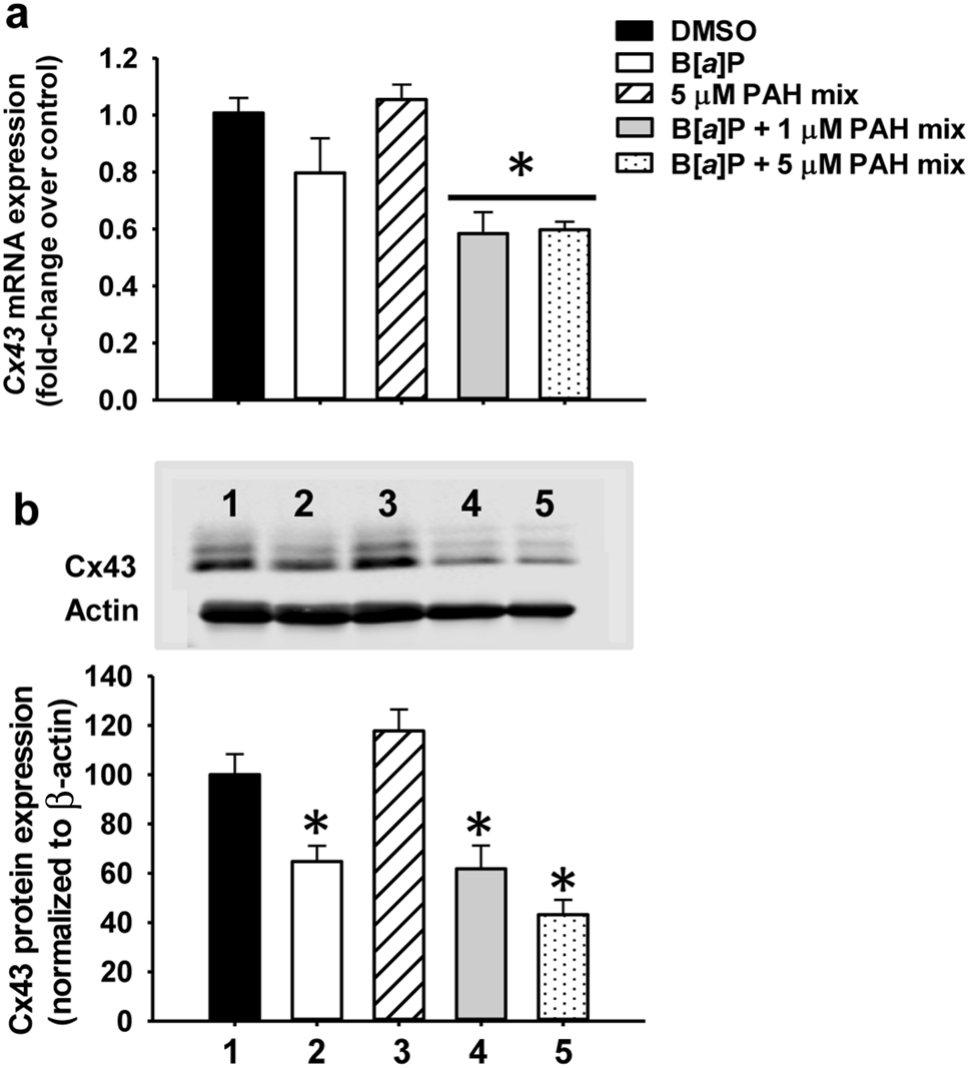
Connexin 43 gene and protein expression in response to B[*a*]P and B[*a*]P in combination with LMW binary PAH mixture. **a** Connexin 43 (Cx43; *Gja1*) mRNA expression in C10 cells treated with 1 µM B[*a*]P, LMW binary PAH mixture (PAH mix) or combinations of 1 µM B[*a*]P and LMW binary PAH mixture at several concentrations via quantitative RT-PCR Sybr green assay normalized to 18S rRNA and presented as fold change over DMSO control. Mean ± SEM presented; *n* = 3, repeated twice. *P < 0.05 compared to DMSO control. **b** Cx43 protein expression determined by immunoblot analysis and quantitated by densitometry. Mean ± SEM presented; *n* = 3, repeated twice. *1* DMSO control; *2* 1 µM B[*a*]P; *3* 5 µM LMW binary PAH mixture (PAH mix); *4* 1 µM B[*a*]P + 5 µM LMW binary PAH mix; *5* 1 µM B[*a*]P + 1 µM LMW binary PAH mixture. *P < 0.05 compared to DMSO control

Further examination of GJIC included evaluating changes in the primary pulmonary connexin involved in gap junction formation, Cx43 (Osgood et al. 2017). Cx43 gene expression (*Gja1*) was not altered in response to 1 µM B[*a*]P or 5 µM LMW binary PAH mixture alone (Fig. 6a). However, Cx43 gene expression was significantly reduced in response to 1 µM B[*a*]P in combination with both 1 and 5 µM LMW binary PAH mixture, demonstrating an additive effect on gene expression. Lastly, 1 µM B[a]P significantly reduced Cx43 protein expression while the 5 µM PAH mixture did not, however, Cx43 protein levels were also reduced with the B[a]P combinations at both 1 and 5 µM LMW PAH mixtures (Fig. 6b). While these Cx43 protein expression levels did not significantly differ between B[*a*]P alone or the combination groups, there was a decreasing trend in Cx43 expression at the B[a]P with 5 µM LMW binary PAH mixture group supporting the reduced GJIC activity and mRNA expression observed at these concentrations (Fig. 6a).

### Cytochrome p4501b1 mRNA expression in response to PAHs

**Fig. 7:**
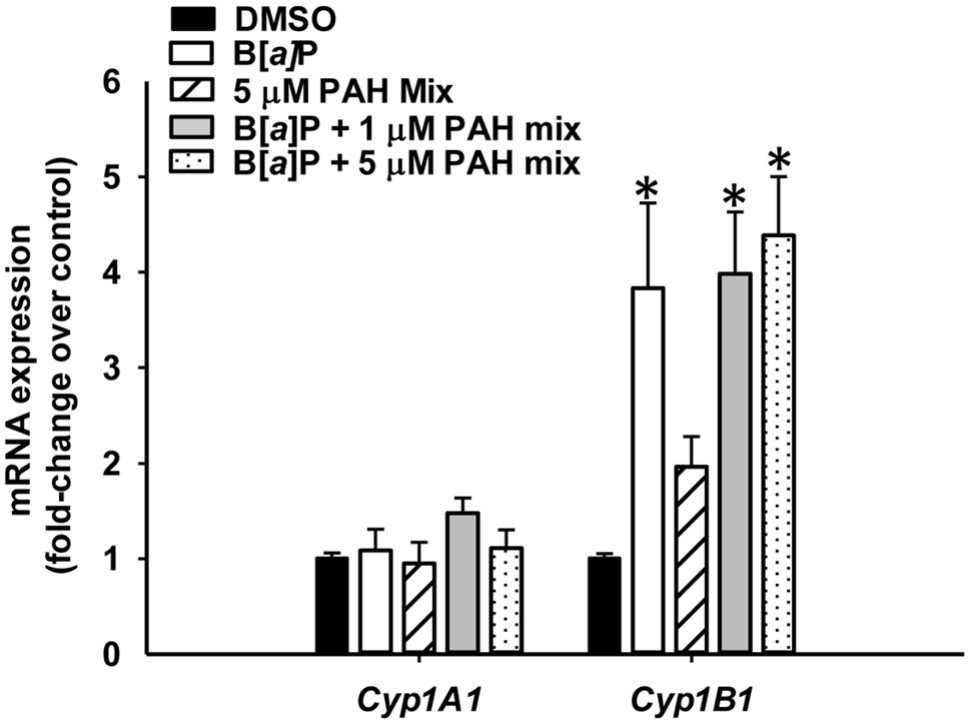
Cytochrome p450 gene expression comparison in C10 cells in response to B[*a*]P and B[*a*]P combinations with the LMW binary PAH mixture. Cytochrome p4501A1 (*Cyp1A1*) and 1B1 (*Cyp1B1*) mRNA expression in C10 cells treated 1 µM B[*a*]P, LMW binary PAH mixture (PAH mix) or combinations of 1 µM B[*a*]P and LMW binary PAH mixture at several concentrations with via quantitative RT-PCR Sybr green assay normalized to 18S rRNA and presented as fold change over DMSO control. Mean ± SEM presented; *n* = 3, repeated twice. *P < 0.05 compared to DMSO control

PAHs are primarily metabolized by cytochrome p450 enzymes (Cyp1a1 and Cyp1B1) to their active metabolites (Miller and Ramos 2001); however, in the C10 cells, prior to 24 h, metabolism is very low (Reiners et al. 1992). Therefore, we tested the *Cyp1a1* and *Cyp1b1* mRNA expression at 24 h of PAH exposure (Fig. 7). Significant increases in *Cyp1b1* mRNA expression were observed in C10 cells treated with 1 µM B[*a*]P and the B[*a*]P combinations with the LMW PAHs, however these three did not differ between groups. Increases in Cyp1b1 mRNA expression was far less pronounced compared to B[*a*]P and the combination experiments with the LMW PAHs alone at the 5 µM dose. Interestingly, there were no changes observed in *Cyp1a1* mRNA expression, similar to our other studies in the human-derived A549 cell line (S. Plöttner, personal communication).

### Induction of the Cox-2 gene in response to PAHs

**Fig. 8:**
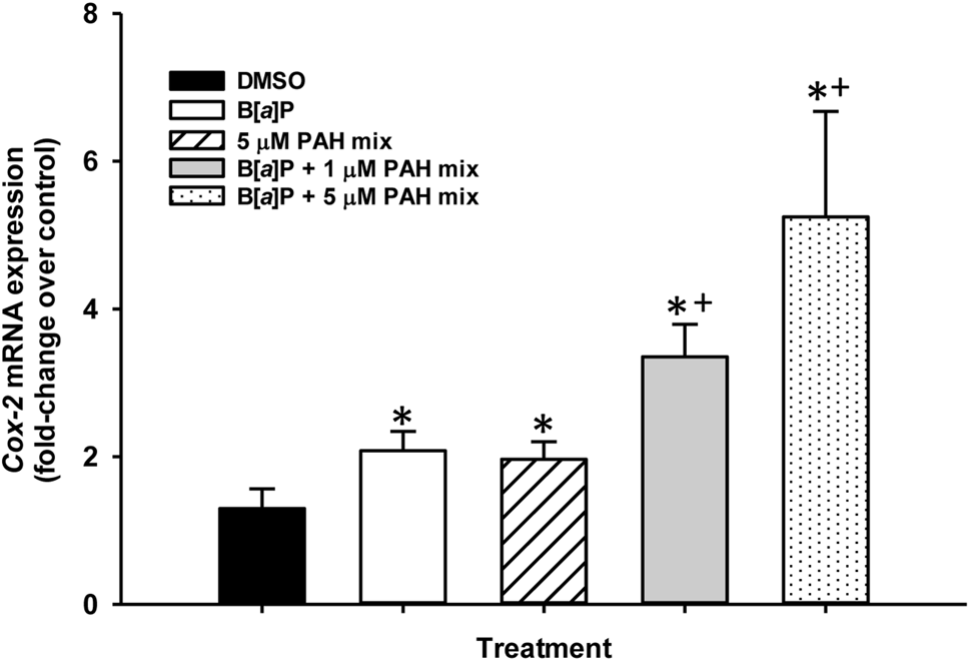
Cyclooxygenase 2 (*Cox-2*) gene expression in response to B[*a*]P and B[*a*]P combinations with the LMW binary PAH mixture. *Cox-2* mRNA expression in C10 cells treated 1 µM B[*a*]P, LMW binary PAH mixture (PAH mix) or combinations of 1 µM B[*a*]P and LMW binary PAH mixture at several concentrations with via quantitative RT-PCR Sybr green assay normalized to 18S rRNA and presented as fold change over DMSO control. Mean ± SEM presented; *n* = 3, repeated twice. *P < 0.05 compared to DMSO control; ^+^ *P* < 0.05 compared to B[a]P and 5 uM PAH mix alone

Due to the involvement of inflammation in the early stages of cancer development and our previous findings in the C10 cells demonstrating increased *Cox-2* (*Ptgs2*) mRNA expression in response to the LMW binary PAH mixture (Osgood et al. 2017), we evaluated *Cox-2* expression at 24 h in the same PAH combinations described above but at 8 and 40 times lower levels as we used previously, 5 and 1 µM PAH mix (Fig. 8). B[a]P and 5 µM LMW binary PAH mixture alone were significantly elevated above that observed with DMSO, however, the combination of B[*a*]P and the LMW binary PAH mixture at both the 1 and 5 µM dose significantly increased expression above that observed in the control treated cells and B[a]P and 5 µM LMW binary PAH mixture alone, further supporting the effects of the combination of PAHs.

## Discussion

Lung ADC is a non-small cell lung carcinoma (NSCLC) subtype that is the most prevalent among both smokers and non-smokers (American Cancer Society 2017) with many etiologies besides smoking including a number of environmental and occupational exposures involving PAHs, such as air pollution, diesel exhaust, coal and coke production exposures, all categorized by the IARC as group 1 carcinogens (IARC 2010, 2012a, b, 2013, 2016). In developing and transition countries the use of indoor coal fueled stoves release high levels of PAHs and is strongly linked to high lung cancer mortality, as well as to the development of other respiratory inflammatory diseases, including chronic obstructive pulmonary disease (COPD) (Zhang and Smith 2007). Diesel exposure, particularly in nonsmokers, also significantly associates with increased risk of lung cancer (Silverman et al. 2012). This current study demonstrates the need for an improved understanding of LMW PAHs that are typically far more abundant in these exposures described above than the HMW PAHs and potentially a re-evaluation of their carcinogenicity following additional in vivo studies and/or in vitro studies in human cell lines.

Since the 1990s, B[*a*]P, a HMW PAH, has been the reference PAH to determine relative potency factors to estimate carcinogenicity and rank toxicity (Nisbet and LaGoy 1992; Environmental Criteria and Assessment; Office 1993), while due to a lack of knowledge, there are many PAHs, particularly those smaller in size, categorized as non-genotoxic, non-carcinogenic, and non-cytotoxic in most models (Ghoshal et al. 1999; Tai et al. 2007; Upham et al. 2008). However, we recently demonstrated that several of these LMW PAHs can elicit numerous adverse lung cell responses in mouse lung cells, namely, induce cytotoxicity, inhibit gap junctions, activate MAP kinases, and induce inflammatory pathways such as cyclooxygenase, Cxcl1 (Kc), and Il6 following acute 30 min–24 h exposures (Osgood et al. 2017). It is also the case that the LMW binary PAH mixtures compared to the individual LMW PAHs did not always respond in an additive manner, but instead in a more synergistic manner, specifically mRNA expression and cytotoxicity (Osgood et al. 2017). This suggests that these PAHs differ in their mechanisms of action (MOA) (Osgood et al. 2017). Importantly, some of these phenotypes are considered key components of the tumor promotion stage of carcinogenicity, specifically, GJIC inhibition, induction of inflammatory mediators, and activation of mitogenic signaling (i.e., MAP kinase pathways), all involved in certain hallmarks of cancer (Hanahan and Weinberg 2011; Klaunig et al. 2000; Trosko and Upham 2010; Zhang and Smith 2007). GJIC is involved in the evasion of growth suppression (Nahta et al. 2015), inflammation in the enabling characteristic of tumor promoting inflammation (Hanahan and Weinberg 2011), and MAP kinase pathways in the self-sufficiency in growth signals (Hanahan and Weinberg 2011). It appears that these LMW PAHs cannot initiate carcinogenesis, however, acting at the promotion stage of cancer or as a co-carcinogen, is still considered as a carcinogen. Thus, in these novel studies we evaluated several established carcinogenic endpoints (DNA adduct formation, GJIC inhibition, and induction of an inflammatory mediator pathway), typically acting during the early stages of carcinogenicity, following exposure of C10 cells to a known classic carcinogen (B[*a*]P) in combination with the same LMW binary PAH mixture we previously used to provide evidence that these LMW PAHs can act as co-carcinogens in the presence of B[*a*]P.

### B[a]P DNA adducts and cancer development

Our studies revealed that both the B[*a*]P DNA adducts and gap junctional activity acted in a manner expected for a carcinogen. DNA adducts such as those observed in our studies, are regarded as a critical step in the initiation stage of carcinogenesis. Anti-BPDE adducts can lead to mutations via transversions such as G:C → T:A, although DNA repair mechanisms can remove and replace these adducts (Miller and Ramos 2001). DNA adducts were significantly increased in the presence of the B[a]P and LMW PAH combination compared to B[*a*]P alone. The fact that these adducts were increased suggests that the LMW PAHs can act as co-carcinogens, however, it does not rule out the potential promoting capability of these LMW PAHs.

### GJIC and cancer development

The significant inhibition of GJIC observed with B[*a*]P combined with LMW PAHs at these low doses compared to B[*a*]P alone further demonstrates that these LMW PAHs act as co-carcinogens. Gap junctions, composed of connexins (Cx), are intercellular channels that allow for molecular communication between neighboring cells that are often inhibited by toxicants, such as tumor promoters (Trosko and Upham 2010). As mentioned above, GJIC is involved in the hallmark of cancer concerning evasion of growth suppression and based on studies in vitro (Cesen-Cummings et al. 1998; Osgood et al. 2017; Tai et al. 2007; Trosko and Upham 2010; Upham et al. 2008) and in vivo (Avanzo et al. 2004), GJIC inhibition is a critical step in the early stages of cancer development. Although controversial, it is hypothesized that connexins act as tumor suppressors that when inhibited, the growth-promoting factors are no longer diluted and intracellular signaling increases which leads to enhanced growth and eventually tumor development (Nahta et al. 2015). For example, Cx43 is significantly reduced in response to multiple toxicants, such as the tumor promoter 12-O-tetradecanoylphorbol-13-acetate (TPA) in both rodent and human epithelial cell types of the lung (C10 cells), liver (WB cells), and breast (MCF-10A cells) (Osgood et al. 2013; Rakib et al. 2010; Upham et al. 2008). Additionally, mice heterozygous for a *Cx43* deficiency developed more lung tumors than their wildtype counterparts (Avanzo et al. 2004) supporting the importance of gap junctions in tumor suppressive activities. Lastly, aberrant *GJA1* mRNA expression was observed in NSCLC patients (Chen et al. 2003). However, other mechanisms exist that could be involved in this underlying mechanism (e.g., hemichannel formation) that need to be explored further (Nahta et al. 2015).

### Inflammation and cancer

The involvement of inflammation in cancer is not a novel concept and is now considered a critical component of cancer development, elucidated by the classification of inflammation as an enabling characteristic in tumor promotion by Hanahan and Weinberg in 2011. In particular, the pathway evaluated herein, COX-2, leads to the production of prostaglandins, such as PGE_2_ and PGF_2_. PGE_2_ can induce tumor progression through epidermal growth factor receptor (EGFR) signaling and increased proliferative responses in lung ADC (Bazzani et al. 2017). COX-2 is also known to be significantly elevated in NSCLC, although clinical trials for COX-2 inhibitors (e.g., apricoxib) have not proven successful as a therapy for lung ADC (Edelman et al. 2017). *Cox-2* was significantly elevated 4–8 h (> 20-fold increase) following treatment with the same LMW binary PAH mixture as used in these studies; however, the doses where these effects were observed were substantially higher (40 µM). The increase observed following 24 h used here at a 8–40-fold lower dose of the LMW binary PAH mixture elicited significant responses with B[*a*]P or the LMW binary PAH mixture alone and also in all of the combinations of B[*a*]P with the LMW binary PAH mixture compared to control, further supporting the co-carcinogenic or tumor promoting capabilities of these LMW PAHs. However, the response was significantly increased over the B[a]P or LMW PAH mixture alone when in combination with either B[*a*]P and 1 or 5 µM PAH mix. Another recent study demonstrated that direct lung application of B[*a*]P in C57BL/6 mice did not elicit inflammation above that observed in the controls (measured via bronchoalveolar lavage analysis)(Arlt et al. 2015), which supports our in vitro results that B[a]P alone is a weak inflammagen.

### PAH exposures and carcinogenicity

Several studies have examined B[*a*]P carcinogenicity in animal models. For example, a skin cancer study at low doses demonstrated that B[*a*]P acted as an initiator, and in the presence of promoters (other LMW PAHs), skin tumors developed (Warshawsky et al. 1993), further supporting the tumor promotion potential of LMW PAHs. Additionally, when coal tar was compared to B[*a*]P alone at the same concentration as observed in the coal tar, B[*a*]P-induced lung tumor numbers were significantly lower and did not equate to the number of tumors observed in the coal tar exposed group (Fitzgerald et al. 2004). Again, the results suggest that B[*a*]P does not act alone in PAH-induced lung carcinogenesis.

A recent paper indicated that different carcinogenic PAHs have different MOAs, via studies using transcriptomics (Labib et al. 2016). Carcinogenic HMW PAHs had different transcript profiles and while they all induced DNA adducts, the MOAs for carcinogenicity were not the same, thus cannot be based on B[*a*]P alone. Therefore, the use of B[*a*]P as the reference PAH may be overestimating or underestimating the carcinogenicity of these PAHs. For the LMW PAHs, based on our previous report and others (Osgood et al. 2017; Upham et al. 2008), it is critical that other endpoints are evaluated for the PAHs that have no initiating effects, but are acting in a co-carcinogenic or tumor promoting manner, or these important early stage cancer responses could be underestimated and overlooked.

### Co-carcinogenic effects of PAHs and toxic equivalency factors

Lastly, to more clearly understand the differences between the complete carcinogenesis endpoints versus co-carcinogenesis and/or promoting endpoints, we assessed the toxic equivalency factors (TEFs) of B[*a*]P, Flthn and 1-MeA for their co-carcinogenic effects. For this analysis, we used GJIC inhibition as the toxicological endpoint of interest in the C10 cells using the 24 h data generated for these studies and our previous studies (Osgood et al. 2017). We also set B[*a*]P as our reference standard (TEF = 1.0). Figures 4a and 5a show that 1 µM B[*a*]P results in approximately 47 and 34% reduction in GJIC (= 53 and 66% fraction of control) which can be averaged to about 40% reduction in GJIC. Our recently published data on the inhibition of GJIC by Flthn and 1-MeA revealed a similar 40% reduction for approximately 10 µM Flthn and 20 µM 1-MeA (Osgood et al. 2017). Therefore, the TEFs for GJIC inhibition in C10 cells following 24 h exposure can be estimated to be approximately 1.0 : 0.1 : 0.05 for B[a]P, Flthn, and 1-MeA with B[a]P showing the highest and 1-MeA showing the lowest GJIC inhibition.

Applying these calculated TEFs to the three mixtures which we have tested (1 µM B[a]P + 1, 5, or 10 µM PAH mix consisting of 1:1 Flthn and 1-MeA) and using an additive model would result in approximately 43, 55, and 70% inhibition of GJIC (= 57, 45, and 30% fraction of control). The actual observed GJIC inhibition in our experiments were 47, 62 and 66% (= 53, 38 and 34% fraction of control, Fig. 5a), respectively, and thus well in agreement with the calculated results.

Overall, our results suggest that the co-carcinogenic effects of PAH mixtures are the sum of the effects caused by the respective individual compounds. In addition, TEFs can be used to assess effects of mixtures such as PAHs. However, the use of TEFs must be endpoint-specific. For example, previously published TEFs which have been established for carcinogenicity [e.g., 1.0 for B[a]P or 0.001 for Flthn; (DFG 2012; Nisbet and LaGoy 1992)] cannot be applied to co-carcinogenic endpoints such as GJIC because they would underestimate these co-carcinogenic effects, in this example by 100-fold. In turn, the TEFs for co-carcinogenic effects cannot be applied to assess the actual carcinogenic outcome of PAH mixtures in animals or humans because they would overestimate carcinogenicity. The molecular endpoints used in our studies reflect those that are known to contribute both to initiating events (DNA adducts) and to co-carcinogenesis or tumor promoting events (GJIC and COX-2) and not meant as endpoints for complete carcinogenesis. This conflation of a carcinogen that is a complete carcinogen versus those that are either co-carcinogens or promoters needs better clarification for future risk assessment. Co-carcinogens and promoters do not result in tumor development unless in the presence of an initiator, such as B[*a*]P, however, both are still carcinogens.

## Conclusions

Many sources of potential exposure to PAHs exist, with the LMW PAHs typically in higher abundance in tobacco smoke, occupational settings, and environmentally such as (urban) ambient air (ATSDR 2005; IARC 2012a, b; Lee et al. 2010), however, most research has focused solely on the HMW PAHs, specifically B[*a*]P, due to IARC categorization. In these studies, we are the first to suggest that LMW PAHs in combination with B[*a*]P can elicit increased carcinogenic potential via increases in BPDE-DNA adducts, inhibition of GJIC, and induction of *Cox-2*. We used an alveolar type II cell line from mouse as a surrogate to determine the actions of these combined PAHs in lung tissue and in the future will use mouse models as well as human cell lines to further validate these findings. Our studies together with others (Labib et al. 2016) support the need for these additional studies to determine if re-evaluation of IARC categorization for these LMW PAHs is warranted due to the likelihood that they are co-carcinogenic or tumor promoters.

## Electronic supplementary material

Below is the link to the electronic supplementary material.

Supplementary material 1 (DOCX 27 KB)
