# Supplementary material for: Environmentally prevalent polycyclic aromatic hydrocarbons can elicit co-carcinogenic properties in an in vitro murine lung epithelial cell model
Source: Arch Toxicol. 2017 Nov 23;92(3):1311–22. doi: 10.1007/s00204-017-2124-5 (PMC5866845; doi:10.1007/s00204-017-2124-5)
Supplement: Supplementary file 1 — Supplementary material 1 (DOCX 27 KB) [file 204_2017_2124_MOESM1_ESM.docx]

**Archives in Toxicology**

**Environmentally prevalent polycyclic aromatic hydrocarbons can elicit co-carcinogenic properties in an *in vitro* murine lung epithelial cell model**

Alison K. Bauer,^1*^ Kalpana Velmurugan^1**^, Sabine Plöttner^2**^, Katelyn J. Siegrist^1^, Deedee Romo^1^, Peter Welge^2^,Thomas Brüning^2^, Ka-Na Xiong^1^and Heiko U. Käfferlein^2^

^1^Department of Environmental and Occupational Health, Colorado School of Public Health, University of Colorado Anschutz Medical Campus, Aurora, CO, 80045, U.S.A.

^2^Institute for Prevention and Occupational Medicine of the German Social Accident Insurance, Institute of the Ruhr-University Bochum (IPA), 44789 Bochum, Germany

*To whom correspondence should be addressed at: [alison.bauer@ucdenver.edu](mailto:alison.bauer@ucdenver.edu)

| **Table S1: Primer sequences for qRTPCR analysis** | | | |
| --- | --- | --- | --- |
| **Name** |  | **Primer Sequence** | **Genbank**  **Accession/NCBI** |
| *18S* | Forward  (5’ 3’) | GAG AAA CGG CTA CCA CAT CCA A | NR_003278 |
|  | Reverse  (5’ 3’) | CCT CCA ATG GAT CCT CGT TAA AG |  |
| *Cx43* | Forward  (5’ 3’) | CAA CGT GGA GAT GCA CCT GAA G | M63801 |
|  | Reverse  (5’ 3’) | GCA CTC AGG CTG AAC CCA TAG A |  |
| *Cyp1a1* | Forward  (5’ 3’) | TGT ATG GAC TTC CAG CCT TC | NM_009992.4 |
|  | Reverse  (5’ 3’) | GTC GGC ACA GTC ACT GTC TA |  |
| *Cyp1b1* | Forward  (5’ 3’) | TGA ATC ATG ACC CAG CCA AGT | NM_009994.1 |
|  | Reverse  (5’ 3’) | ATT GCA CTG ATG AGC GAG GAT |  |
| *Ptgs2* | Forward  (5’ 3’) | AAA CCG TGG GGA ATG TAT GAG C | NM_011198.4 |
|  | Reverse  (5’ 3’) | TCG CAG GAA GGG GAT GTT GTT C |  |
